# Halogen‐Bonded Hole‐Transport Material Enhances Open‐Circuit Voltage of Inverted Perovskite Solar Cells

**DOI:** 10.1002/advs.202411567

**Published:** 2024-10-22

**Authors:** Zhaoyang Chen, Jiakang Zhang, Zilong Chen, Ze‐Fan Yao, Kai‐Kai Liu, Zhongmin Zhou, Haichang Zhang, Maning Liu

**Affiliations:** ^1^ Key Laboratory of Rubber‐Plastics of Ministry of Education/Shandong Province (QUST), School of Polymer Science and Engineering Qingdao University of Science and Technology 53‐Zhengzhou Road Qingdao 266042 P. R. China; ^2^ College of Chemistry and Molecular Engineering Qingdao University of Science and Technology Qingdao 266042 P. R. China; ^3^ College of Chemistry and Molecular Engineering Peking University Beijing 100871 P. R. China; ^4^ Centre for Analysis and Synthesis, Department of Chemistry Lund University Lund 22100 Sweden; ^5^ Wallenberg Initiative Materials Science for Sustainability, Department of Chemistry Lund University Lund 22100 Sweden; ^6^ NanoLund Lund University Lund 22100 Sweden

**Keywords:** halogen bonding, hole‐transport materials, interfacial charge recombination, inverted structure, open‐circuit voltage

## Abstract

Interfacial properties of a hole‐transport material (HTM) and a perovskite layer are of high importance, which can influence the interfacial charge transfer dynamics as well as the growth of perovskite bulk crystals particularly in inverted structure. The halogen bonding (XB) has been recognized as a powerful functional group to be integrated with new small molecule HTMs. Herein, a carbazole‐based halo (iodine)‐functional HTM (O1), is synthesized for the first time, demonstrating a high hole mobility and suitable energy levels that align well with those of perovskites. The strong interaction between O1 and perovskite, i.e., I···I^−^, induces the formation of an ordered interlayer, which are verified by both theoretical and experimental studies. Compared to the reference HTM (O2) without any halo‐function, the XB‐induced interlayer effectively enhances the interfacial charge extraction efficiency, while significantly hindering the non‐radiative charge recombination by reducing the surface traps upon the strong passivation effect. This is reflected as a big increase in the open‐circuit voltage by up to 114 mV in the fabrication of inverted devices with the highest power conversion efficiency of 22.34%. Moreover, the ordered XB‐driven interlayer at the interface of O1 and perovskite is mainly responsible for the extended lifespan under the operational conditions.

## Graphical abstract

A new carbazole‐based halo (iodine)‐functional small molecule (O1) is successfully synthesized and employed as a hole‐transport material (HTM) in inverted perovskite solar cells. Compared to the reference O2 HTM without any halo‐function, the strong interaction between O1 and perovskite, i.e., I···I^‐^ halogen bonding, leads to a big increase by 114 mV in the open‐circuit voltage of corresponding devices.

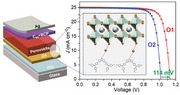

## 1 Introduction

Inverted perovskite solar cells (IPSCs) with a p‐i‐n structure have attracted rising attention owing to their comparably high efficiency with standard n‐i‐p structure, negligible hysteresis, low temperature process, relatively high stability and good compatibility with flexible substrates.^[^ ^1^, ^2^, ^3^, ^4^, ^5^, ^6^, ^7^, ^8^, ^9^ ^]^ To achieve high‐performance IPSCs, the hole‐transport material (HTM) layer plays a significant role not only in the efficient hole transfer after the charge separation at the interface of HTM and perovskite, but also in the formation of high quality atop perovskite thin film (bulk crystals) as the growth template.^[^ ^10^, ^11^, ^12^ ^]^ Thus, the interfacial properties of HTM and perovskite are of high importance to be fine‐tuned from both performance and stability points of view. The surface passivation has been considered as one effective strategy to tailor the interfacial properties, leading to the suppression of non‐radiative charge recombination on the perovskite surface as well as the protection of perovskite layer against the penetration of water and oxygen molecules.^[^ ^13^, ^14^, ^15^, ^16^ ^]^ Instead of insertion of a separate interlayer that triggers the passivation effect at the HTM and perovskite interface, the integration of functional groups with the design of new small molecule HTMs has been recently emphasized, due to the two‐in‐one effect within an HTM that also simplifies the fabrication process. Several representative functional groups have been included in the HTMs, which could bind to the undercoordinated Pb cations such as O‐Pb, N‐Pb and hydrogen bonding.^[^ ^17^, ^18^, ^19^ ^]^ On the other hand, the coordination with iodide anions upon the non‐covalent interactions has been rarely investigated, with a classic example of halogen bonding (XB).

XBs are particularly attractive due to their directionality, adjustable interaction strength, and common hydrophobic nature.^[^ ^20^, ^21^ ^]^ Benefiting from these unique properties, XBs have been widely used in soft functional matters and crystal tuning, for instance, in designing supramolecules and liquid crystals or synthesizing photo‐responsive polymers.^[^ ^22^, ^23^, ^24^ ^]^ In halide perovskite precursors, iodide anions, which act as Lewis bases, can strongly interact with halogenated organic molecules via non‐covalent interactions, leading to the formation of XBs.^[^ ^25^, ^26^ ^]^ These XBs are similar to the well‐known hydrogen bonding, which could be used for interfacial toughening, crystallization manipulation, and trap passivation in perovskite solar cells. Numerous studies have highlighted the key effect of XB on the perovskite crystal structure of perovskite, resulting in a more symmetrical surface.^[^ ^27^, ^28^, ^29^ ^]^ Additionally, XB tends to guide the formation of ordered and structured layers, significantly enhancing both device performance and stability. These findings suggest that XB represents a rational supramolecular strategy for improving the interfacial properties of perovskites.

In a recent study, Canil et al. developed a carbazole‐based material, namely PFI, that could act as an iodine‐containing HTM, anchoring to the perovskite surface via XBs.^[^ ^30^ ^]^The perovskite surface was well functionalized through the iodine‐iodine interaction at the interface, resulting in the suppressed carrier recombination and improved charge extraction in the application of standard perovskite solar cells. Zhang et al. proposed the bidentate and undercoordinated iodine on the perovskite surface to construct stable perovskite‐based heterostructures.^[^ ^31^ ^]^ This strong XB effectively hindered the interfacial migration of iodide ions toward adjacent functional layers such as C60 and Ag in a standard structure. Very recently, Bie et al. designed a series of halogenated HTMs containing Br and Cl atoms, which induced a strong passivation effect on the surface of quasi‐2D perovskite in an inverted structure, achieving a highest efficiency of 21.07%; however still mainly based on the interactions with undercoordinated Pb ions, i.e., Br‐Pb^2+^ or Cl‐Pb^2+^.^[^ ^32^ ^]^ To the best of our knowledge, there is no report on iodine‐based XB that is integrated with an HTM to directly interact with the perovskite surface in an inverted structure.

In this work, a long iodine‐based haloalkane chain was first designed to anchor on the carbazole‐based core to form a new small molecule HTM, namely O1, which possesses a high hole mobility, suitable energy levels, and most importantly the capability to initiate the formation of I···I^−^ XB between O1 and perovskite in an inverted structure. We also synthesized a reference HTM (O2) with the almost identical structure and properties only except for the absence of iodine atom in the structure thus no formation of XB is expected. Our theoretical (density functional theory, DFT) and experimental (X‐ray photoelectron spectroscopy, XPS) results clearly confirmed the formation of strong XB‐based interlayer at the interface of O1 and perovskite, leading to the enhanced charge extraction while significantly hindering the non‐radiative charge recombination compared to the reference case of O1. This improvement eventually contributed to a big increase in the open‐circuit voltage (*V*_oc_) by 114 mV in the fabrication of high‐performance IPSCs, which demonstrated the highest power conversion efficiency (PCE) of 22.34%. Moreover, the operation stability of O1‐based devices was considerably improved with a projected T_80_ lifetime of >1000 h, which benefits from the effective protection of XB‐induced interlayer at the interface area.

## 2 Results and Discussion

### 2.1 Design and Synthesis of O1 and O2 Molecules

**Scheme 1:**
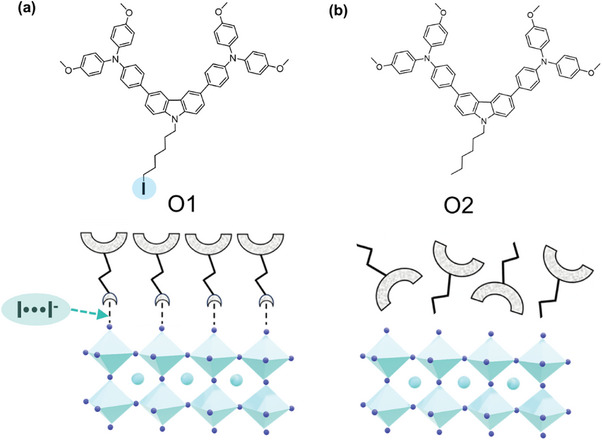
Chemical structures of a) O1 and b) O2 with corresponding hypothesized XB functionalization on the perovskite surface.

The halogenated units in most of reported HTMs are often located on the aromatic rings,^[^ ^33^, ^34^, ^35^ ^]^ which is conducive to the charge extraction and transfer within the molecules. However, the high steric hindrance of an aromatic ring makes it difficult for halogen atoms to flexibly interact with the perovskite surface, which hardly form an effective XB. In this work, we have designed a carbazole‐based halogenated molecule (C_58_H_54_IN_3_O_5_, namely O1) with a long alkyl chain of iodohexane, which is easily capable of anchoring to the perovskite surface via XB, i.e., I···I^−^ (**Scheme** **1**a), due to the σ‐vacancy that is generated by the intramolecular iodine atoms.^[^ ^36^ ^]^ A comparable molecule (C_58_H_55_N_3_O_5_, namely O2) has been designed as the reference (Scheme 1b) with the same structure and properties except for the lack of an iodine atom, leading to no formation of XB at the perovskite interface. O1 and O2 molecules were synthesized via a facile method with the detailed routes (see Schemes S1–S3, Supporting Information) in the Supporting Information. Our results of ^1^H and ^13^C NMR confirm the successful formation of O1 and O2 molecules in desired structures (Figures S1–S7, Supporting Information). The synthetic costs (≈$30 g^−1^, see the cost analysis in the Supporting Information) of O1 and O2 HTMs are much lower than those of currently commercial HTMs such as PTAA ($423 g^−1^) and Spiro‐OMeTAD ($92 g^−1^),^[^ ^37^, ^38^ ^]^ which is promising for future commercialization. The thermal stability was investigated for O1 and O2 molecules by measuring the thermogravimetric analysis (TGA) curves, as shown in Figure S8 (Supporting Information). Both HTM molecules exhibit relatively good thermal stability, with a 5% weight loss observed at 291 and 279 °C, respectively. As expected, O1 demonstrates even better resistance to thermal induced decomposition compared to O2, possibly due to the adjacent halogen‐bonded dipole interaction forms a dimer‐like material within O1. Based on density functional theory (DFT) calculations, both O1 and O2 dimers indeed exhibit an ordered π‐π stacking, while O1 dimer has a stronger intermolecular interaction force (see Figure S9, Supporting Information). By calculating the dipole moment changing from the ground state to excited state, the optically generated dipoles have been estimated. It is noted that O1 dimer demonstrates a larger dipole interaction induced by the ending iodine atoms, and therefore the dipole‐dipole interaction forms a drift field, which could be beneficial for observed high thermal stability as well as charge transport within the HTM.^[^ ^39^ ^]^

### 2.2 Optical, Electrochemical and Hole‐Transport Properties

**Figure 1:**
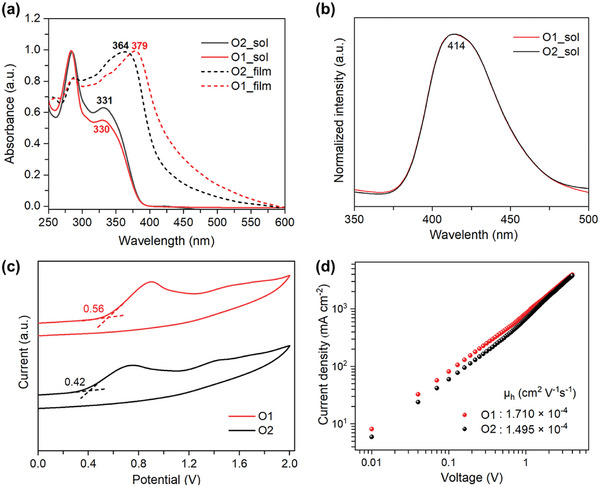
a) UV–vis absorption spectra of O1 and O2 HTMs both in solution and film phases. b) Normalized photoluminescence (PL) spectra of O1 and O2 molecules in solution, excited at 330 nm. c) Cyclic voltammetry (CV) spectra of O1 and O2 films on ITO glasses as working electrode in acetonitrile with the tetrabutylammonium hexafluorophosphate (TBAPF_6_, 0.1 M) as the supporting electrolyte. The oxidation potential was calculated versus ferrocene. d) Dark *J*–*V* curves of hole‐only devices with structure “ITO/PEDOT: PSS/HTM/Ag”.

**Figure** **1**a depicts the comparison of UV‐vis absorption spectra of O1 and O2 molecules both in solution and in film phases. A clear red shift in the first exciton peak position has been observed for both O1 (≈49 nm) and O2 (≈33 nm) molecules when changing from the solution phase to the film state. This suggests that more pronounced interaction between the iodine atoms during the formation of O1 molecules compared with the case of O2, could result in an enhanced aggregation and more orderly film deposition. In general, a strong aggregation in HTM film is accompanied by close molecular interaction, which could extend the effective conjugated system, as evidenced by our X‐ray diffraction (XRD) data (Figure S10, Supporting Information). The XRD pattern of O1 film exhibits more distinguishable peak at a lower Bragg angle of ≈17° when comparing to that (≈21°) of O2 film.^[^ ^40^, ^41^ ^]^ The relatively weak crystal orientation has been observed in the XRD patterns of both O1 and O2 HTM films that were prepared via spin‐coating method, which normally produces an intermediate phase between amorphous and highly crystalline phases.^[^ ^39^, ^40^ ^]^ Due to the identical backbone structure of O1 and O2 molecules, both show almost same photoluminescence (PL) spectra, centered at 414 nm (see Figure 1b). Based on the absorption onsets of these films, the optical bandgaps of O1 and O2 HTMs were (estimated to be 2.92 and 3.00 eV. Additionally, cyclic voltammetry (CV) measurements were performed to determine the oxidation potentials (E_onset(ox)_) for O1 (0.56 V) and O2 (0.42 V) (Figure 1c), which were converted to the highest occupied molecular orbital (HOMO, E_HOMO_) levels of O1 (−5.36 eV) and O2 (−5.22 eV) HTMs based on the equation presented in **Table** **1**. Considering the valence band (≈−5.60 eV) of the perovskite layer in this work, O1 HTM shows a better energy alignment with a suitable gap of ≈240 meV between HOMO level of O1 and valence band of perovskite, as evidenced by the minimized voltage loss of corresponding devices that will be discussed in later section. All optical and electrochemical properties of O1 and O2 HTMs are summarized in Table 1.

**Table 1:** Optical, electrochemical, and hole‐transport properties of O1 and O2 HTMs.

HTM	λ_abs,exc_ [nm]		HOMO^a^ ^)^ [eV]	LUMO^b)^ [eV]	E_g_^opt^ ^c)^ [eV]	µ_h_ ^d)^ [cm^2^ V^−1^ s^−1^]
	Solution	Thin film				
O1	283	364	−5.36	−2.44	2.92	1.71 × 10^−4^
O2	284	379	−5.22	−2.22	3.00	1.49 × 10^−4^

^a)^ HOMO level was obtained by CV measurement: ‐E_HOMO_ = E_onset(ox)_ + 4.8 eV, where E_onset(ox)_ is the onset potential for the oxidation versus ferrocene;

^b)^ LUMO (lowest unoccupied molecular orbital) level was obtained from the equation of E_LUMO_ = E_HOMO_ + E_g_^opt^;

^c)^ Optical bandgap Eopt g was determined at the absorption onset of the molecule in solution phase (E_g_^opt^ = 1240/λ_abs@onset_ eV);

^d)^ Hole mobility.

As one key factor for high‐performance devices, the hole mobilities of HTMs were measured by space charge limited current (SCLC) method.^[^ ^42^ ^]^ Figure 1d shows a comparison of the dark *J*‐*V* ^2^ curves of the hole‐only devices with structure “ITO/PEDOT:PSS/HTM/Ag”. The Mott‐Gurney equation of µ = $\frac{8J_{D}L^{3}}{9\varepsilon_{0}\varepsilon_{r}V^{2}}$ was used to determine the hole mobilities (µ_h_) of O1 and O2 HTMs, where ε_0_ is the vacuum dielectric constant, ε_r_ is the relative dielectric constant (for organic materials, ε_r_ = 3), L is the thickness of the HTM layer, *J*_D_ is the dark current density, and *V* is the applied voltage. The O1 HTM indeed exhibits a higher mobility (1.71 × 10^−4^ cm^2^ V^−1^ s^−1^) than the O2 HTM (1.49 ×10^−4^ cm^2^ V^−1^ s^−1^) (see Table 1), indicating that the capacity of hole transport could be effectively enhanced upon the formation of iodine induced dipole moment within O1 dimers.

### 2.3 Simulated XB at the Interface of O1 and Perovskite

**Figure 2:**
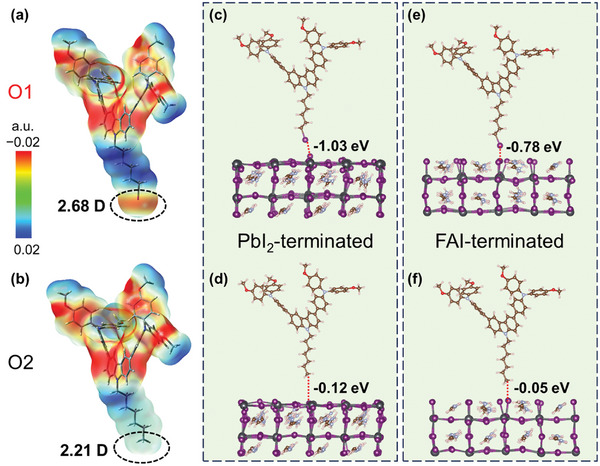
Electrostatic potential distribution of single a) O1 and b) O2 molecules. The dash cycle highlights the potential area of dipole. D is the unit of dipole, i.e., Debye. Density functional theory (DFT) simulations of the adsorption of O1 (top) and O2 (bottom) HTMs on FAPbI_3_ perovskites with c,d) PbI_2_‐terminated surface and e,f) FAI‐terminated surface, respectively. The dotted line highlights the adsorption energy in each case.

To first gain insight into the intramolecular charge delocalization of halogenated HTM, we conducted the electrostatic potential analysis for single O1 (**Figure** **2**a) and O2 (Figure 2b) molecules. It was found that the positive charges delocalized on the O atoms of TPA moieties as well as on the alkyl chains anchoring on the carbazole units for both O1 and O2 HTMs. The dominant negative charges accumulated on the N atoms of TPA moieties and carbazole cores in both cases, while a narrow negative charge accumulation area was only detected on the iodine atom of O1 molecule. This indicates that the extra charge distribution induced by CH_2_‐I in O1 molecule contributes to the enhancement of dipole moment (2.68 Debye) compared to that (2.21 Debye) of O2 molecule based on the DFT calculations. We in turn simulated the interaction of O1 and O2 with the perovskite surface by using FAPbI_3_ as the prototype of perovskite in this work, where FA is methylammonium. As shown in Figure 2c–f, two situations are identified for the FAPbI_3_ surface, i.e., PbI_2_‐terminated and FAI‐terminated, by establishing a model on the (001) surface of FAPbI_3_ in the supercell method.^[^ ^43^, ^44^, ^45^ ^]^The detailed DFT calculation method is described in the SI. Regardless of the termination of the perovskite surface, O1 HTM demonstrates much stronger interaction with the perovskite surface by showing significantly higher adsorption energies by almost one order of magnitude than those of O2 HTM. This clearly suggests that the XB indeed forms at the interface of O1 and perovskite upon the strong I∙∙∙I^–^ interaction, leading to the formation of an oriented interlayer on the perovskite surface due to the directional nature of XB.^[^ ^46^, ^47^ ^]^ On the other hand, there is no presence of well‐defined interaction between O2 HTM and perovskite layer, as evidenced by the simulated low adsorption energies. In this case, O2 HTM behaves a random and uneven interaction with the perovskite surface by showing much longer bonding (C∙∙∙I) distances (≈6.43 Å in the case of PbI_2_‐terminated surface) compared to those (≈ 3.05 Å) of I∙∙∙I between O2 HTM and perovskite. Our DFT calculations theoretically support the hypothesis of the formation of strong XB between O1 HTM and perovskite, which could significantly influence the interfacial properties.

### 2.4 Influence of XB on Interfacial Properties

**Figure 3:**
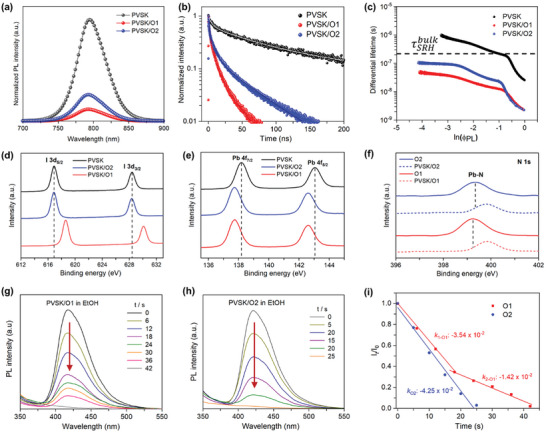
a) PL spectra and b) time‐resolved PL (TRPL) decays (excited at 473 nm from glass side) of glass/perovskite (PVSK), glass/O1/perovskite (PVSK/O1), and glass/O2/perovskite (PVSK/O2) respectively. Solid lines in b) represent the best fitting for the plots of differential lifetimes in c). c) Differential lifetime (*τ*_PL_) versus the logarithm of the corresponding PL intensity (ln(*ϕ*PL)). The plateau (dash line) of *τ*_PL_ highlighted for the control sample (PVSK) was used to calculate the SRH lifetime as $\tau_{SRH}^{bulk}$. XPS spectra of perovskite films without and with HTMs in structure of glass/HTM/PVSK for d) I and e) Pb elements. XPS spectra of HTM films with and without capping with perovskite films for f) N element. PL spectra of g) glass/PVSK/O1 and h) glass/PVSK/O2 samples after immersion in ethanol for different time intervals. i) Comparison of the change in intensity (I_t_) of PL peaks from g) and h) depending on the immersing time (*t*), where I_0_ is the initial PL intensity at immersion time *t* = 0. The change rate (*k*) of PL peak intensity is highlighted at different stages.

To determine the effect of XB functionalization on the interfacial charge extraction process in an inverted structure, we first performed the steady‐state photoluminescence (PL) measurements for glass/perovskite (control), glass/O1/perovskite, and glass/O2/perovskite samples. **Figure** **3**a depicts the PL spectra of these three samples excited at 473 nm from the glass side to avoid the excitation of O1 and O2 HTM themselves. The change in the PL intensity, i.e., emission quenching, could reflect the charge transfer efficiency at the interface of perovskite and charge selective layer.^[^ ^48^ ^]^ The quenching efficiency (also known as hole extraction efficiency) can be estimated by comparing the PL amplitude of a glass/ HTM/perovskite sample with that of a glass/perovskite sample. Both O1 and O2 coated on the perovskite films clearly quenched the PL emission of the pristine perovskite film, indicating good hole extraction ability. Sequentially, O1 (89%) showed a more efficient quenching efficiency compared to O2 (74%). To further investigate the influence of as‐formed XB induced interlayer on the interfacial charge transfer dynamics, we then measured the time‐resolved PL (TRPL) decays of these same samples, as compared in Figure 3b. As desired, the glass/O1/perovskite sample demonstrates a more accelerated PL decay compared to the O2 case, suggesting that the XB‐driven interlayer at the interface of O1 and perovskite indeed promotes the hole extraction kinetics. To quantitatively analyze the PL decays, the differential lifetime (τ_PL_) for each PL decay was extracted based on Equation (1):

(1) $$\tau_{\mathrm{PL}}={\left(-\frac{1}{m}\frac{d\;\ln\left(\phi_{\mathrm{PL}}\right)}{dt}\right)}^{-1}$$

where ϕ_PL_(*t*) presents the PL intensity at time *t* after the excitation and the *m* presents a factor that is related to the extraction level, which is 2 by assuming the perovskite is intrinsic.^[^ ^49^ ^]^ The calculated results were plotted as differential lifetime versus PL intensity in logarithm ($\ln\left(\phi_{\mathrm{PL}}\right)$) in Figure 3c. The effective Shockley Read Hall (SRH) lifetime both in the bulk (without HTM, $\tau_{SRH}^{bulk}$) and with HTM ($\tau_{SRH}^{HTM}$) can be determined as 207.5 ns (pristine perovskite), 11.4 ns (with O1), and 23.3 ns (with O2), respectively. We further calculated the surface recombination velocity (*S*), particularly at the interface of HTM and perovskite based on Equation (2):

(2) $$\tau_{SRH}^{HTM}={\left(\frac{1}{\tau_{SRH}^{bulk}}+\frac{S}{2d}\right)}^{-1}$$

where *d* presents the perovskite layer thickness (≈ 600 nm according to the cross‐sectional scanning electron microscopy (SEM) image shown in following section). The glass/O1/perovskite sample exhibits a high *S* of 9948 cm s^−1^ that is more than twice higher than the case (4572 cm s^−1^) of O2, indicating that the XB‐initiated interlayer could significantly reduce the surface traps at the perovskite and O1 interface while effectively hindering the interfacial charge recombination, as evidenced by the enhanced hole extraction efficiency.

To provide direct evidence of XB formation at the interface of perovskite and O1 HTM, we conducted the X‐ray photoelectron spectroscopy (XPS) measurements on pristine perovskite films (glass/perovskite), pristine HTM films (glass/HTM) and blending films (glass/HTM/perovskite). As observed in Figure 3d, the I 3*d* peak of glass/O1/perovskite film clearly shifts to a higher binding energy compared to the reference case of pristine perovskite film, while there is no obvious shift of I 3*d* peak for the glass/O2/perovskite film. We attribute the I 3*d* peak shift toward higher binding energy in the case of glass/O1/perovskite, to the decreased electron density of iodide anions (I^−^) on the surface of perovskite as the Lewis base, which donates the electron to the I atom in a state of σ‐hole^[^ ^20^ ^]^ in the O1 HTM. This undoubtfully suggests that the strong I∙∙∙I^–^ interaction between O1 and perovskite triggers the formation of XB, which is absent at the interface of O2 and perovskite. Additionally, the as‐formed XB dominates the passivation effect at the interface of O1 and perovskite, due to all other common peaks (i.e., Pb 4*f*, N 1*s* and O 1*s*) show similar shifts in the binding energy in both cases of O1 and O2 when compared with the reference of pristine perovskite film (see Figure 3e,f; Figure S11, Supporting Information). To further confirm the robustness of the XB interaction between O1 and perovskite, we conducted a solvent exposure test by dipping the glass/perovskite/HTM samples (O2 as reference) in ethanol for several seconds that can gradually dissolve the materials. After each time of dipping, the PL spectra of samples were immediately measured by monitoring a range of 350–550 nm that only corresponds to the HTMs emission until no PL can be observed (i.e., HTM film is fully dissolved). To further determine the effect of XB at the interface area, we conducted a solvent test^[^ ^30^ ^]^ by immersing the glass/perovskite/HTM films in a polar solvent (i.e., ethanol) and measuring the PL spectra at interval times by taking out the samples. Figure 3g,h show the evolution of PL spectra of perovskite/O1 and perovskite/O2 samples after immersion in ethanol with different interval times. The change rate (*k*) of PL peak intensity is defined as I*_t_*/I_0_ (I*_t_* is the PL peak intensity at dipping interval time and I_0_ is the initial PL peak intensity), as plotted in Figure 3i. Interestingly, the profile of O1‐based sample demonstrates a clear inflection point after immersing in ethanol for 18 s, indicating a slower dissolving speed while there is no change in the PL decreasing rate during the whole dissolution process of O2‐based sample in ethanol. Therefore, we attribute this kink point in the case of O1‐based sample to the strong interaction of XB formed at the perovskite and O1 interface, which makes a good resilience to the solvent dissolution as a protective layer.

### 2.5 Performance of Halogen‐Bonded IPSCs

**Figure 4:**
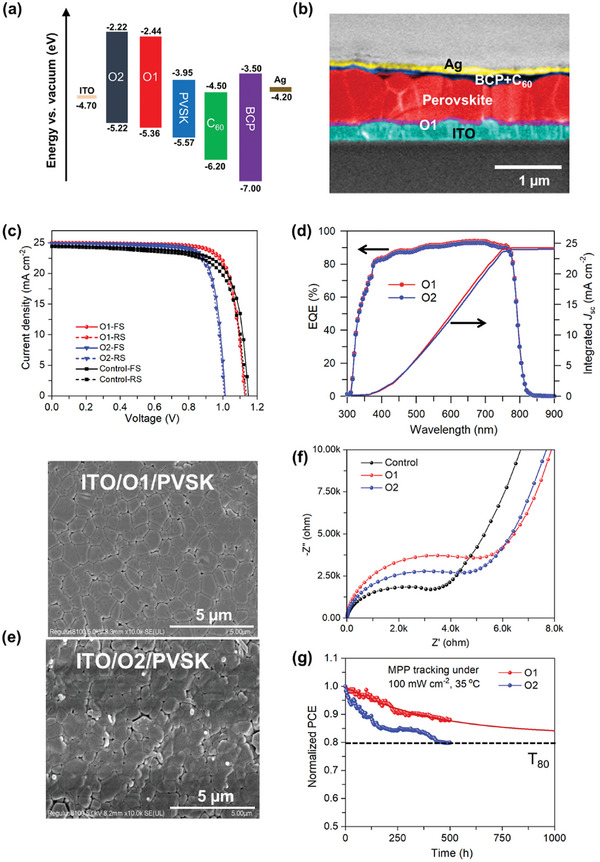
a) Energy level diagram of each layer of IPSCs. b) Cross‐sectional SEM image of a typical O1‐based IPSC with estimated thicknesses of 600 nm for the perovskite layer and 50 nm for the O1 HTM layer, respectively. c) *J*–*V* curves of the champion PTAA (control), O1, and O2‐based IPSCs under 1 sun condition (100 mW cm^−2^), scanned from the forward direction. d) EQE spectra and integrated *J*_sc_ of champion devices presented in (c). e) Morphological SEM images of O1 (top panel) and O2 (bottom panel)‐based glass/HTM/PVSK films. The scale bar is 5 µm. f) Electrochemical impedance spectroscopy (EIS) Nyquist plots of PTAA (control), O1, and O2‐based IPSCs. g) Normalized PCEs of encapsulated O1 and O2‐based IPSCs in air and under light soaking at the MPP (1 sun illumination, 100 mW cm^−2^; 420 nm UV light cut‐off filter; 35 °C). The solid line represents the projection of T_80_ lifetime up to 1000 h.

To assess the photovoltaic performance of undoped O1 and O2 HTMs, we fabricated p‐i‐n IPSCs with structure “ITO/HTM/PVSK/C_60_/BCP/Ag”. **Figure** **4**a presents the energy level diagram of each layer in a typical device. It is noted that the O1 HTM features a deeper (140 meV) HOMO level compared to that of O2 HTM, leading to a less thermodynamic loss when aligning with the valence band of perovskite.^[^ ^50^ ^]^ thickness of HTM layer was first optimized by achieving the best device performance, which is ≈50 nm for O1 HTM according to the cross‐sectional SEM image in Figure 4b. The commercial HTM, i.e., PTAA, was used to fabricate the reference devices (control). Figure 4c depicts the current density (*J*)–voltage (*V*) curves of champion IPSCs with different HTMs under 1 sun condition (100 mW cm^−2^) and both forward and reverse scans. Interestingly, both O1 and O2‐based devices exhibit similar photogenerated current densities, i.e., 25.04 mA cm^−2^ for O1 and 24.93 mA cm^−2^ for O2, which can be verified by their integrated *J*_sc_ in the external quantum efficiency (EQE) spectra in Figure 4d. The steady‐state output test (see Figure S12, Supporting Information) demonstrates the stable power output within 300 s for all devices. One key photovoltaic parameter that dominates the device performance is the open‐circuit voltage (*V*_oc_), while the O1‐based devices show a significant enhancement by up to 114 mV compared to that of O2‐based devices. This suggests that the strong passivation effect upon the as‐formed XB‐induced interlayer on the interface of perovskite and O1 indeed hinders the charge recombination by significantly reducing the shallow traps. To confirm the trap density at the interface of perovskite and HTM, we fabricated hole‐only devices with structure ITO/PEDOT:PSS/PVSK/HTM/Ag, which were measured with their dark *J–V* curves, as shown in Figure S13 (Supporting Information). The calculated trap density (*N*_t_ = 0.272 × 10^15^ cm^−3^) of O1‐based device is effectively lower than that (0.319 × 10^15^ cm^−3^) of O2‐based one, which makes good agreement with enhanced *V*_oc_ and surface recombination velocity in the case of O1 HTM.

It is noteworthy that the hysteresis index (0.011) of O1‐based devices is much lower than that (0.031) of control devices (PTAA) while even the O2‐based devices exhibit moderately low hysteresis index (0.018). This clearly indicates that the hole swiftly transfers by departing from the XB‐tailored interface of O1 and perovskite after the hole extraction, and then the built‐in electric field reduces at the interface.^[^ ^51^ ^]^ All the photovoltaic parameters of as‐fabricated IPSCs are summarized in **Table** **2**. Moreover, the interfacial XB interlayer in the case of O1 HTM could also influence the growth of atop perovskite bulk crystals in the inverted structure, as shown in the morphological SEM images in Figure 4e and f. The ITO/O1/perovskite film exhibits more homogeneous morphology and high quality of compact perovskite thin film with less grain boundaries compared to that of ITO/O2/perovskite film with low homogeneity and more pinholes particularly on the grain boundaries, which could contribute to the enhancement in both performance of stability of O1‐based IPSCs. Even compared to the PTAA‐based reference devices, the overall performance (22.34% PCE) of O1‐based devices demonstrates higher PCEs by 6% enhancement, ascribed to the improved fill factor (FF) that benefits from the XB interlayer triggered decrease in the series resistance, as evidenced by the electrochemical impedance spectra (EIS) in Figure 4g. The O1‐based devices show the highest recombination resistance (*R*_re_) compared to other two cases (O2 and PTAA), which make good agreement with observed suppression of interfacial charge recombination. On the other hand, the observed series resistance (R_s_) of O1‐based devices is slightly higher than that of O2‐based ones, which may result in a small decrease in the FF as shown in Table 2. This is plausibly due to a bit less equilibrium between the charge transfer (hole and electron transfer) at interfaces of O1‐based devices compared to the O2 case, which will be investigated in our future work. It is worth noting that the EIS profiles do not show the closed semi‐cycles instead by upward tails, possibly due to the charge transfer processes at interfaces (e.g., electron transfer from the perovskite layer to the electron transport layer or recombination at defects) may not fully equilibrate at low frequencies, leading to an upward tail in the Nyquist plot.

**Table 2:** Performance of p‐i‐n IPSCs fabricated with different HTMs based on 16 devices in each case. All data are recorded from the forward scan.

HTM	*V*_oc_ [V]	*J*_sc_ [mA cm^−2^]	FF [%]	PCE [%]	HI^b)^
PTAA (control)	1.140±0.010 (1.149)^a^ ^)^	24.45±0.12 (24.52)	74.96±0.44 (75.64)	21.01±0.21 (21.31)	0.031
O2	1.010±0.010	24.85±0.11	79.45±0.32	19.97±0.17	0.018
	(1.014)	(24.93)	(79.81)	(20.18)	
O1	1.120±0.010	24.97±0.08	78.8±0.35	22.13±0.17	0.011
	(1.128)	(25.04)	(79.10)	(22.34)	

^a)^ The values in brackets correspond to the photovoltaic parameters of champion cells;

^b)^ HI represents hysteresis index of best devices, which equals to (PCE_FS_‐PCE_RS_)/PCE_FS_. FS is forward scan and RS is reverse scan.

We in turn qualified the effect of XB‐initiated interlayer on the operation stability of encapsulated O1‐based devices by measuring the maximum power point tracking (MPPT, see Figure 4h) under light soaking (1 sun illumination, 100 mW cm^−2^; 420 nm UV light cut‐off filter; 35 °C). The O1‐based devices show much higher stability with an estimated T_80_ lifetime of >1000 h compared to that (T_80_: ≈500 h) of O2 case, attributed to not only the effective protection (as observed high solvent resistance) of XB interlayer on the interface of perovskite and O1, but also the higher quality of as‐formed perovskite layer in inverted structure. Furthermore, the surface wettability of pristine HTM films was tested by measuring the water contact angles (WCA) of these films on glass (see Figure S14, Supporting Information). The O1 film possesses a higher WCA (74°) than that (63°) of O2 film, indicating a better hydrophobicity for the former case, which is likely due to the greater polarity of iodine atom that is connected to the side chain in the O1 molecular structure.

## 3 Conclusion

In summary, we have designed and synthesized a new carbazole‐based halo‐functional small molecule (O1) as the efficient HTM in the application of IPSCs. Compared to the reference HTM (O2) without any halogenated atoms, a strong and directional I···I^−^ bonded interlayer has formed at the interface of O1 and perovskite, as evidenced by both DFT calculation and XPS data. This XB‐driven interlayer significantly improves the interfacial charge transfer properties with higher charge extraction efficiency and suppressed non‐radiative carrier recombination. Moreover, the ordered XB interlayer at the interface of O1 and perovskite acts as a good template for the growth of high‐quality perovskite thin film with larger bulk crystal size and less grain boundaries compared to the case of O2. All these merits from the formation of XB‐based interlayer eventually contributed to a distinguishable enhancement in the *V*_oc_ by 114 mV of O1‐based IPSCs than the reference devices, resulting in a high PCE of 22.34% that is one of the highest records in small molecule HTM‐based IPSCs. More importantly, the hydrophobic nature of XB together with the self‐protection of XB‐induced interlayer demonstrates an excellent resilience to solvent exposure as well as to water penetration, leading to an improved operational stability of O1‐based devices with an estimated T_80_ lifetime of >1000 h, which is almost twice as that (≈500 h) of the reference O2‐based devices. This work demonstrates an effective strategy that through alkyl‐chain engineering the introduction of iodine atom into the small‐molecule HTMs is promising for high‐performance and stable IPSCs due to the interfacial interaction via I···I^−^ XB. Regarding to the new design of halo‐functional HTMs, the efforts might be made on the modification of molecular backbone or the units of side conjugated chains.

## Conflict of Interest

The authors declare no conflict of interest.

## Supporting information



Supporting Information

## Data Availability

The data that support the findings of this study are available from the corresponding author upon reasonable request.
