# Supplementary material for: Halogen‐Bonded Hole‐Transport Material Enhances Open‐Circuit Voltage of Inverted Perovskite Solar Cells
Source: Adv Sci (Weinh). 2024 Oct 22;11(46):2411567. doi: 10.1002/advs.202411567 (PMC11633543; doi:10.1002/advs.202411567)
Supplement: Supplementary file 1 — Supporting Information [file ADVS-11-2411567-s001.pdf]

ADVANCED SCIENCE
Open Access

Supporting Information

for *Adv. Sci.*, DOI 10.1002/advs.202411567

Halogen-Bonded Hole-Transport Material Enhances Open-Circuit Voltage of Inverted Perovskite Solar Cells

Zhaoyang Chen, Jiakang Zhang, Zilong Chen, Ze-Fan Yao, Kai-Kai Liu, Zhongmin Zhou, Haichang Zhang* and Maning Liu**

Supporting Information

Halogen-Bonded Hole-Transport Material Enhances Open-Circuit Voltage of Inverted Perovskite Solar Cells

Zhaoyang Chen,¶ Jiakang Zhang,¶ Zefan Yao, Zilong Chen, Kaikai Liu, Zhongmin Zhou, Haichang Zhang,* and Maning Liu**

*Corresponding authors: maning.liu@chem.lu.se (M. Liu);

haichangzhang@qust.edu.cn (H. Zhang); zhouzm@qust.edu.cn (Z. Zhou)

¶These authors equally contribute to this work.

Contents

1. Experimental section 3
1.1 Materials 3
1.2 Synthesis of molecules 3
1.2.1 Synthesis of TPA-Bo 3
1.2.2 Synthesis of O2 4
1.2.3 Synthesis of O1 5
1.2.4 Synthetic cost analysis for O1 and O2 HTMs 6
1.3 Preparation of perovskite precursor 10
1.4 Fabrication of IPSCs 10
1.5 Characterization 11
1.5.1 Structure Characterization. 11
1.5.2 Electrochemical Measurements 11
1.5.3 Thermal properties characterization 11
1.5.4 UV/vis absorption spectra 11
1.5.5 DFT calculations 11
1.5.6 Other characterization 12
2. Results 13
2.1 NMR spectra 13
2.2 TGA spectra 16
2.3 Simulated dimer arrangement 17
2.4 XRD patterns of HTM films 17
2.5 XPS spectra of HTM/perovskite films 18
2.6 Steady-state output test 18
2.7 Dark *J-V* curves of hole-only devices 19
2.8 WCAs for HTMs coated on glasses 19
References 19

1. Experimental section

1.1 Materials

All reagents were purchased from commercial sources and used without further purification unless otherwise noted. 4-Bromoaniline, KOH, CuI, 1,10-phenanthroline, 6-bromohexanol, 1-bromohexane, 3,6-dibromocarbazole, 1,6-diiodohexane, tert-butanol, diethyl succinate, 4-dimethylaminopyridine (DMAP), di-tert-butyl dicarbonate, $Pd(PPh_3)_4$, K_2CO_3, KOAc, [1,1'-bis(diphenylphosphino)ferrocene] dichloropalladium(II), and $Pd(PPh_3)_4$ were obtained from Energy Chemical.

1.2 Synthesis of molecules

1.2.1 Synthesis of TPA-Bo

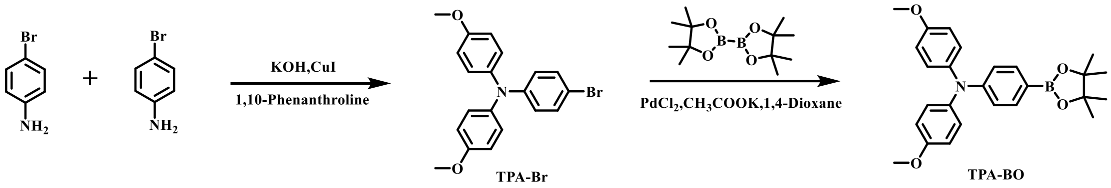

Scheme S1. Synthetic route of TPA-BO.

4-Bromo-*N,N*-bis(4-methoxyphenyl)aniline (TPA-Br)

5-Bromoaniline (2 g, 11 mmol), p-iodoanisole (6.8 g, 29 mmol), KOH (5.2 g, 92 mmol), CuI (0.38 g, 1 mmol), and 1,10-phenanthroline (0.42 g, 2 mmol) were added into freshly distilled toluene (90 ml) under N_2 protection. Subsequently, the reaction mixture was refluxed for 24 hours. After cooling the solution to room temperature, it was extracted with dichloromethane and deionized water three times and dried over anhydrous $MgSO_4$. The crude product was purified by column chromatography (neutral aluminum oxide, dichloromethane: petroleum ether = 1:2) to afford compound TPA-Br (3.25 g, yield: 72%) as a light yellow solid. ^1H NMR (500 MHz, $CDCl_3$) δ: 7.21–7.24 (q, 4H), 7.01–7.03 (q, 4H), 6.80–6.83 (q, 4H), 6.77–6.79 (d, 2H). ^{13}C NMR (500 MHz, $CDCl_3$) δ: 156.05, 147.94, 140.55, 131.77, 126.58, 121.98, 114.78, 112.35, 99.99, 55.50.

4-Methoxy-N-(4-methoxyphenyl)-N-(4-(4,4,5,5-tetramethyl-1,3,2-dioxaborolan-2-yl)phenyl)aniline (TPA-Bo)

TPA-Br (2 g, 5.2 mmol), bis(pinacolato)diboron (1.586 g, 6.2 mmol), KOAc (1.53 g, 15.6 mmol), and [1,1'-bis(diphenylphosphino) ferrocene] dichloro palladium(II) (0.2 g, 0.27 mmol) were added into freshly distilled 1,4-dioxane (20 mL). The mixture was refluxed for 24 hours under N_2 protection. After cooling the mixture to room temperature, it was extracted with dichloromethane and deionized water, then dried over anhydrous $MgSO_4$. The crude product was purified by column chromatography (silica gel, EtOAc: petroleum ether = 1:30) to afford compound TPA-Bo (1.27 g, yield: 56%) as a white solid. ^{1}H NMR (500 MHz, d_1-$CHCl_3$) δ ppm: 7.59-7.60 (d, 2H), 7.04-7.08 (q, 4H), 6.85-6.87 (d, 2H), 6.83 (m, 2H), 6.82 (m, 2H). ^{13}C NMR (500 MHz, d_1-$CHCl_3$) δ ppm: 156.17, 151.36, 140.39, 135.74, 127.11, 126.38, 118.61, 114.79, 83.43, 55.45, 24.87, 24.83.

1.2.2 Synthesis of O2

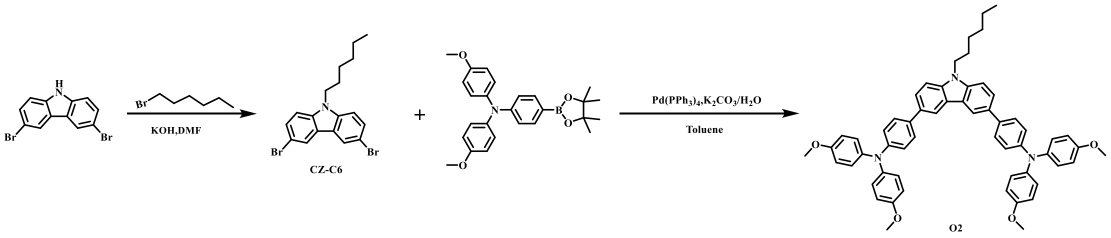

Scheme S2. Synthetic route of O2.

3,6-Dibromo-9-pentyl-9H-carbazole (CZ-C6)

3,6-Dibromocarbazole (1 g, 3 mmol), KOH (0.7 g, 12 mmol), and 1-bromohexane (0.76 g, 4.6 mmol) were added to freshly distilled N,N-Dimethylformamide (DMF) (20 mL) under N_2 protection. The reaction was then refluxed at 120°C for 24 h. After cooling to room temperature, the mixture was extracted three times with dichloromethane and deionized water. Subsequently, the mixture was dried over anhydrous $MgSO_4$. The crude product was purified by column chromatography (silica gel, dichloromethane: petroleum ether = 2:1) to obtain the white solid product CZ-C6 (0.99 g, yield: 82%). ^{1}H NMR (500 MHz, $CDCl_3$) δ: 8.02 (d, J = 1.8 Hz, 2H), 7.44 (dd,

J = 8.7, 1.9 Hz, 2H), 7.15 (d, J = 8.7 Hz, 2H), 4.11 (t, J = 7.2 Hz, 2H), 1.77–1.61 (m, 2H), 1.32–1.10 (m, 6H), 0.84–0.69 (m, 3H). ^{13}C NMR (500 MHz, $CDCl_3$) δ ppm: 139.31, 129.01, 123.45, 123.26, 111.93, 110.39, 43.36, 31.51, 28.83, 26.89, 22.52, 13.99.

4,4'-(9-Hexyl-9H-carbazole-3,6-diyl)bis(N,N-bis(4-methoxyphenyl)aniline) (O2)

CZ-C6 (0.345 g, 0.84 mmol), TPA-Bo (0.8 g, 1.85 mmol), and potassium carbonate solution (2M/L, 2 mL) were added to freshly distilled toluene (6 mL). The mixture was degassed with nitrogen, followed by the addition of $Pd(PPh_3)_4$ (0.049 g, 0.05 mmol). Under N_2 protection, the reaction was carried out at 100°C for 24 hours. The solution was cooled to room temperature, extracted twice with dichloromethane and deionized water, and dried over anhydrous $MgSO_4$. The crude product was purified by column chromatography (silica gel, dichloromethane: ethyl acetate = 1:1), yielding O2(0.48 g, yield: 64%) as a yellow solid. ^1H NMR (500 MHz, $CDCl_3$) δ: 8.30 (s, 2H), 7.68 (d, J = 8.4 Hz, 2H), 7.55 (d, J = 8.3 Hz, 4H), 7.43 (d, J = 8.5 Hz, 2H), 7.10 (s, 11H), 6.86 (d, J = 8.5 Hz, 9H), 4.32 (s, 2H), 3.82 (s, 12H), 1.97–1.82 (m, 2H), 1.33 (qd, J = 7.0, 5.7, 3.1 Hz, 6H), 0.88 (td, J = 5.0, 4.5, 2.2 Hz, 3H). ^{13}C NMR (500 MHz, $CDCl_3$) δ ppm: 155.68, 140.00, 132.05, 127.64, 126.36, 124.83, 123.46, 121.43, 118.25, 114.66, 108.94, 55.51, 43.31, 31.61, 29.06, 27.02, 26.92, 22.58, 14.05.

1.2.3 Synthesis of O1

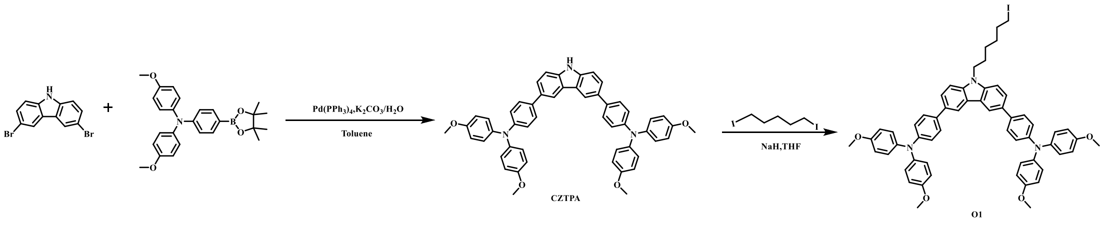

Scheme S3. Synthetic route of O1.

4,4'-(9H-Carbazole-3,6-diyl)bis(N,N-bis(4-methoxyphenyl)aniline) (CZTPA)

3,6-Dibromocarbazole (1.5 g, 1.4 mmol), TPA-Bo (0.47 g, 3.4 mmol), and potassium carbonate solution (2M/L, 9 mL) were added to freshly distilled N,N-dimethyl-

acetamide (20 mL). The mixture was degassed with nitrogen, followed by the addition of $Pd(PPh_3)_4$ (0.083 g, 0.05 mmol). Under N_2 protection, the reaction was carried out at 100°C for 24 h. The solution was cooled to room temperature, extracted twice with dichloromethane and deionized water, and dried over anhydrous $MgSO_4$. The crude product was purified by column chromatography (silica gel, dichloromethane: ethyl acetate = 2:1), yielding CZTPA (0.74 g, yield: 67%) as a white solid. Due to the moderate solubility of the product, the ^{13}C NMR spectrum was not measured. ^1H NMR (400 MHz, $CDCl_3$) δ: 8.25 (s, 2H), 7.63 (d, J = 8.2 Hz, 2H), 7.53 (s, 6H), 7.09 (s, 10H), 6.86 (d, J = 8.2 Hz, 10H), 3.82 (s, 12H).

4,4'-(9-(5-Iodopentyl)-9H-carbazole-3,6-diyl)bis(N,N-bis(4-methoxyphenyl)aniline) (O1)

CZTPA (0.5 g, 0.65 mmol) was added to freshly distilled tetrahydrofuran (12 mL), and NaH (0.1 g, 4.2 mmol) was slowly added to the solution, which was then left at room temperature for 1 h. The mixture was then added to the tetrahydrofuran solution of 1,6-diiodohexane (0.64 mL, 3.87 mmol) through a constant pressure funnel within ten minutes. After stirring for another 2 h, the reaction mixture was extracted three times with dichloromethane and deionized water, then dried over anhydrous $MgSO_4$. The crude product was purified by column chromatography (silica gel, dichloromethane: petroleum ether = 1:1), and the light yellow solid product O1 (0.42 g) was obtained. ^1H NMR (500 MHz, $CDCl_3$) δ: 8.30 (d, J = 1.7 Hz, 2H), 7.68 (dd, J = 8.5, 1.8 Hz, 2H), 7.58–7.50 (m, 4H), 7.42 (d, J = 8.5 Hz, 2H), 7.18–6.99 (m, 12H), 6.92–6.78 (m, 8H), 4.33 (t, J = 7.0 Hz, 2H), 3.82 (s, 12H), 3.15 (t, J = 6.9 Hz, 2H), 1.93 (t, J = 7.0 Hz, 2H), 1.79 (t, J = 7.0 Hz, 2H), 1.52–1.39 (m, 4H). ^{13}C NMR (500 MHz, $CDCl_3$) δ ppm: 155.67, 139.95, 132.15, 127.64, 126.35, 124.89, 123.49, 121.40, 118.27, 114.67, 108.91, 77.47, 77.05, 76.63, 55.52, 33.27, 30.27, 28.93, 26.28, 6.99.

1.2.4 Synthetic cost analysis for O1 and O2 HTMs

Table S1. Materials quantities and cost for the synthesis of **TPA-Br**.

Chemical	Amount g/ml	Price of Chemical $/(g/ml)	Chemical Cost $	Target product $/g
4-Bromoaniline	2	0.065	0.13	**2.011**
p-iodoanisole	6.8	0.214	1.45	
CuI	0.38	0.51	0.19	
KOH	5.2	0.081	0.42	
1,10-phenanthroline	0.42	0.358	0.15	
Toluene	50ml	4.9/500ml	0.49	
$MgSO_4$	4	0.0042	0.0168	
CH_2Cl_2	300ml	0.83/500ml	0.498	
Petroleum ether	300ml	0.72/500ml	0.432	
Aluminum oxide	200	0.0138	2.76	

Table S2. Materials quantities and cost for the synthesis of **TPA-Bo**.

Chemical	Amount g/mL	Price of Chemical $/(g/ml)	Chemical Cost $	Target product $/g
TPA-Br	2	2.011	4.022	**7.36**
KOAc	1.53	0.053	0.08	
$PdCl_2$[DPPF]	0.2	16.3	3.26	
bis(pinacolato)diboron	1.586	0.016	0.0253	
1,4-dioxane	20ml	4.88/500ml	0.195	
$MgSO_4$	4	0.0042	0.0168	
CH_2Cl_2	500ml	0.83/500ml	0.83	
Petroleum ether	500ml	0.72/500ml	0.72	
Silica gel	100	0.002	0.2	

Table S3. Materials quantities and cost for the synthesis of **CZ-C6**.

Chemical	Amount g/ml	Price of Chemical $/(g/ml)	Chemical Cost $	Target product $/g
3, 6-dibromocarbazole	1	0.27	0.27	**1.6258**
KOH	0.7	0.08	0.056	
1-Bromohexane	0.76	0.07	0.053	
DMF	20ml	15.17/500ml	0.6	
$MgSO_4$	4	0.0042	0.0168	
CH_2Cl_2	200ml	0.83/500ml	0.33	
Petroleum ether	200ml	0.72/500ml	0.29	
Silica gel	50	0.002	0.01	

Table S4. Materials quantities and cost for the synthesis of **O2**.

Chemical	Amount g/ml	Price of Chemical $/(g/ml)	Chemical Cost $	Target product $/g
TPA-BO	0.8	7.36	5.888	**24.643**
CZ-C6	1	1.6258	1.6258	
K_2CO_3	2.76	0.0132	0.036	
$Pd(PPh_3)_4$	0.049	56.51	2.768	
Toluene	6ml	4.9/500ml	0.058	
$MgSO_4$	4	0.0042	0.0588	
CH_2Cl_2	400ml	0.83/500ml	0.664	
Petroleum ether	400ml	0.72/500ml	0.576	
Silica gel	100	0.002	0.2	

Table S5. Materials quantities and cost for the synthesis of **CZTPA**.

Chemical	Amount g/ml	Price of Chemical $/(g/ml)	Chemical Cost $	Target product $/g
TPA-BO	1.5	7.36	11.04	**24.0969**
3, 6- dibromocarbazole	0.47	0.27	0.1269	
K_2CO_3	2.76	0.0132	0.036	
$Pd(PPh_3)_4$	0.083	56.51	4.69	
N,*N*-Dimethylacetamide	20ml	15.17/500ml	0.6	
$MgSO_4$	4	0.0042	0.0588	
CH_2Cl_2	400ml	0.83/500ml	0.664	
Petroleum ether	400ml	0.72/500ml	0.576	
Silica gel	100	0.002	0.2	

Table S6. Materials quantities and cost for the synthesis of **O1**.

Chemical	Amount g/ml	Price of Chemical $/(g/ml)	Chemical Cost $	Target product $/g
CZTPA	0.5	24.0969	12.048	**31.3**
NaH	0.1	0.0552	0.00552	
1,6-diiodohexane	0.64	0.9875	0.245	
tetrahydrofuran	12ml	10.206/500ml	0.6	
$MgSO_4$	4	0.0042	0.0168	
CH_2Cl_2	400ml	0.83/500ml	0.33	
Petroleum ether	400ml	0.72/500ml	0.29	
Silica gel	100	0.002	0.02	

1.3 Preparation of perovskite ($FA_{0.8}Cs_{0.17}MA_{0.03}Pb(I_{0.97}Br_{0.03})_3$) precursor

The 1.35 M $FA_{0.9}Cs_{0.07}MA_{0.03}Pb(I_{0.92}Br_{0.08})_3$ perovskite precursor was prepared by dissolving 591.2 mg of PbI_2, 190.4 mg of FAI, 39.6 mg of $PbBr_2$, 24.6 mg of CsI, 13.5 mg of FABr and 6.4 mg of MAI in 1 mL co-solvent of DMSO and DMF (v/v, 4/1). The perovskite precursor solution was stirred on a 60°C heating plate in a nitrogen atmosphere glove box for 6 hours, and then filtered with a 0.22 um filter for standby.

1.4 Fabrication of inverted perovskite solar cells (IPSCs)

The indium-doped tin oxide (ITO) glass substrates with an optical transmission of >80% in the visible range and a sheet resistance of 8−10 Ω^{-2} were purchased from Techno Print Co., Ltd., Japan. The patterned ITO substrate was firstly cleaned using a surfactant, then washed with sequential sonication in deionized water, ethanol and acetone for 10 min, respectively. Finally, it was subjected to UV/ozone treatment for 30 min before utilization. The optimized HTM precursor solution (20 mg/mL in chlorobenzene) was spin-coated at 3000 rpm for 30 seconds, and the HTM film was subsequently annealed at 100 °C and 180 °C for 20 minutes and 10 minutes, respectively. For the perovskite layer, 60 μL of the as-prepared perovskite precursor solution was first dropped onto the as-prepared HTM film, followed by spin-coating at 1000 rpm for 10 seconds and at 4000 rpm for 30 seconds to form the perovskite film. Fifteen seconds before the end of the second spin-coating step, 600 μL of ether was swiftly added to the perovskite film. The film was then transferred to a hot plate and preheated at 75 °C for 2 minutes, followed by further heating at 120 °C in a dry air environment (RH ~30%) for 15 minutes for annealing. After the formation of the perovskite film, the electron transport layer of C_{60} (~30 nm) and the hole-blocking layer of bathocuproine (BCP) (~10 nm) were sequentially evaporated. Finally, a 100 nm thickness of Ag electrode was deposited on top of the substrate using the thermal evaporation method.

1.5 Characterization

1.5.1 Structure Characterization

Nuclear magnetic resonance (NMR) spectra were obtained using a Mercury 500 spectrometer. Elemental analysis was performed using a Carlo Erba 1106 Elemental Analyzer. Infrared spectra were recorded on a Nicolet-6700 Fourier transform infrared (FT-IR) spectrometer by casting films on KBr plates from solution.

1.5.2 Electrochemical Measurements

Cyclic voltammetry (CV) analysis of both polymers was measured by a three-electrode cell with tetra-n-butyl-ammonium hexafluorophosphate ($TBAPF_6$, 0.1 m in acetonitrile) as the supporting electrolyte. Two platinum wires were used as counter electrode and reference electrode and an indium-doped tin oxide (ITO) glass casted with oligomer films was used as the working electrode. The scan rate is 100 mV/s, and all the potentials were corrected to the ferrocene/ferrocene$^+$ (Fc/Fc^+) standard under room temperature.

1.5.3 Thermal properties characterization

Thermalgravimetric analysis (TGA) were performed under nitrogen atmosphere (20 mL/min) using a Netzsch TGA (209F1) (heating rate: 10 °C/min).

1.5.4 UV/vis absorption spectra

UV/vis absorption spectra were recorded using a dual-beam grating Agilent Cary 5000 absorption spectrometer. The thin-film UV/Vis absorption spectra of the three molecules were measured using spin-coated thin films (5 mg/mL solutions of O1 and O2 in tetrahydrofuran, spin-coated onto quartz glass substrates at a rotation speed of 1500 rpm).

1.5.5 DFT calculations

Density-functional theory (DFT) calculations were performed to optimize the geometries and evaluate frontier molecular orbitals of all of molecules. DFT calculations of these finite molecular structures were conducted using the ORCA[1] while the structures were first optimized with GFN2-xtb method, [2] and then further optimized using B3LYP-D3/def2-SVP method to evaluate the electrostatics potential

(ESP) map. Stacked dimers of these molecules were built and optimized using B3LYP-D3/def2-SVP method to evaluate the interaction energies. To understand the interactions between molecules and perovskite surface, DFT calculations with periodic boundary conditions (PBC) were performed. Crystal structure of perovskite ($FAPbI_3$) was used to build the PbI_2-teminated slabs with a 2×6×4 supercell due to the large size of the molecules. A vacuum layer of ~45 Å was added onto the PbI_2-teminated surface to avoid self-interaction. Then, one molecule was put into a box with infinite PBC structures, leading to a vacuum layer of at least ~15 Å. The slabs were optimized unless the maximum geometry change was less than 0.0015 Å. All DFT calculations with PBC were conducted using the CP2K version 2023.1 with xtb method.[3,4] The binding energy, $E_{binding}$, was calculated from $E_{binding} = E_{slab} - (E_{perovskite} + E_{molecule})$. Multiwfn was employed to make the input files. [5,6] VESTA was employed to visualize the simulated structures.[7]

1.5.6 Other characterization

Scanning electron microscope (SEM) images were obtained using a field-emission scanning electron microscope (S-4800, Hitachi). in film X-ray diffraction (XRD) experiments operating at 3 kW were performed on a Powder X-ray Diffractometry (INCA Energy, Oxford Instruments). The films were prepared by drop-coasting of polymer solution (5 mg/ml in chloroform). Steady-state photoluminescence (PL) and time-resolved photoluminescence (TRPL) spectra were obtained using a Delta Flex fluorescence spectrometer (HORIBA). X-ray photoelectron spectroscopy (XPS) measurements were conducted with 150 W monochromatic Al Kα (1486.6 eV) radiation on a Thermo Scientific ESCA Lab 250 Xi. Curve fitting was performed using the XPSPEAK software, with the curves corrected based on the C1s peak at 284.8 eV. Current density-voltage (J–V) curves were characterized using a Keithley 2400 source meter under standard AM 1.5G illumination with a solar simulator (PV Measurements Inc.), equipped with a 450 W Xenon lamp (Newport Corp.) and an output intensity of 100 mW cm^{-2}. The intensity was calibrated with a reference Si cell at the measurement location. The external

quantum efficiency (EQE) spectra were measured using a Sciencetech SF150 xenon arc lamp and a PTI monochromator, with the monochromatic light intensity calibrated by a Si photodiode (Newport, 818-UV). Electrochemical impedance spectroscopy (EIS) curves were obtained using an electrochemical workstation (CHI660E) in the dark, with a frequency range between 0.1 and 1×10^5 Hz.

2. Results

2.1 NMR spectra

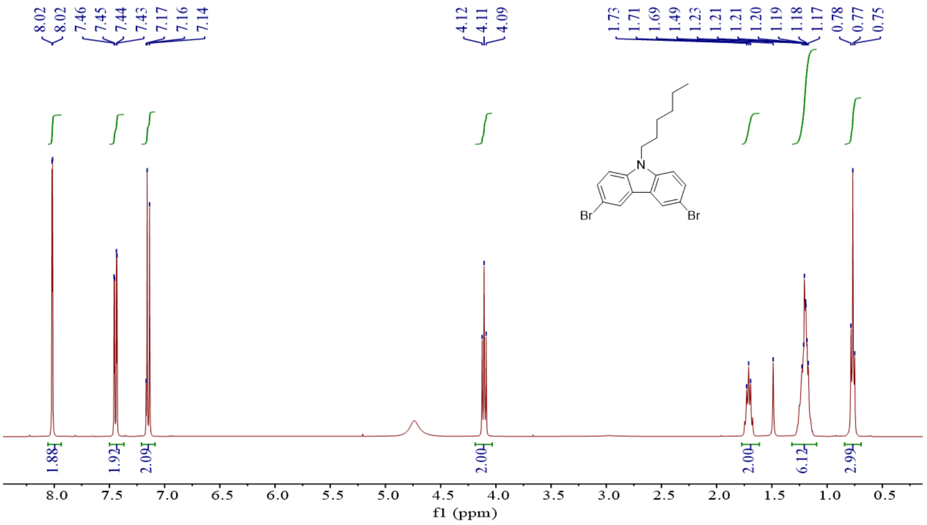

Figure S1. ^{1}H NMR spectra of CZ-C6.

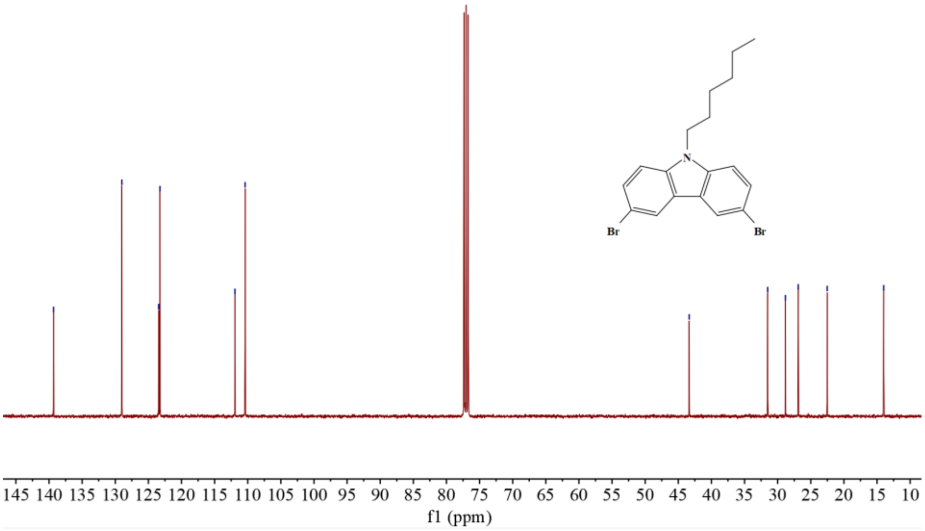

Figure S2. ^{13}C NMR spectra of CZ-C6.

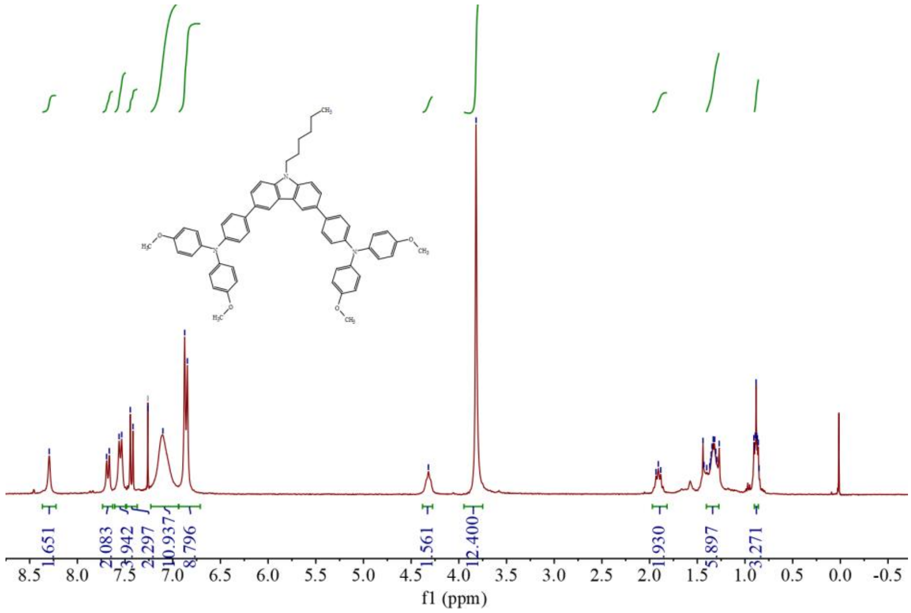

Figure S3. ^{1}H NMR spectra of O2.

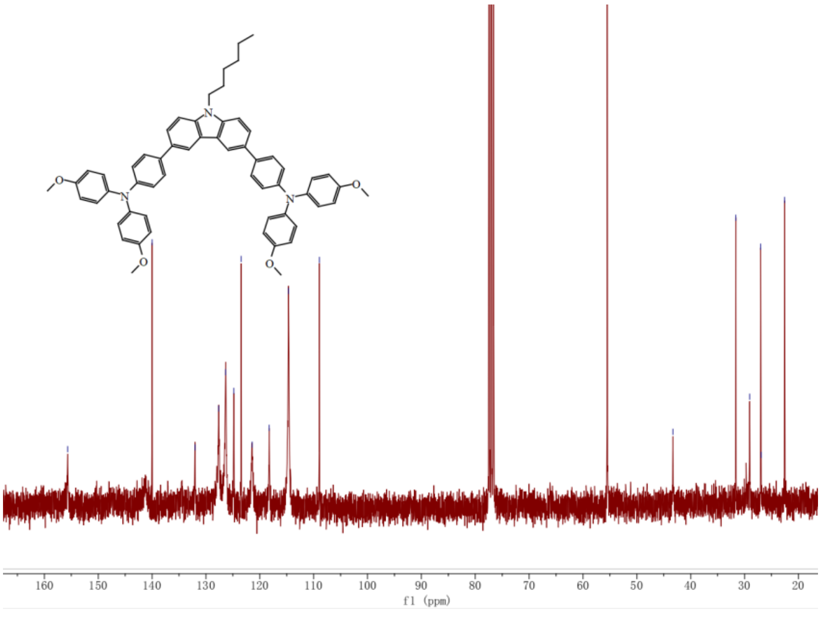

Figure S4. ^{13}C NMR spectra of O2.

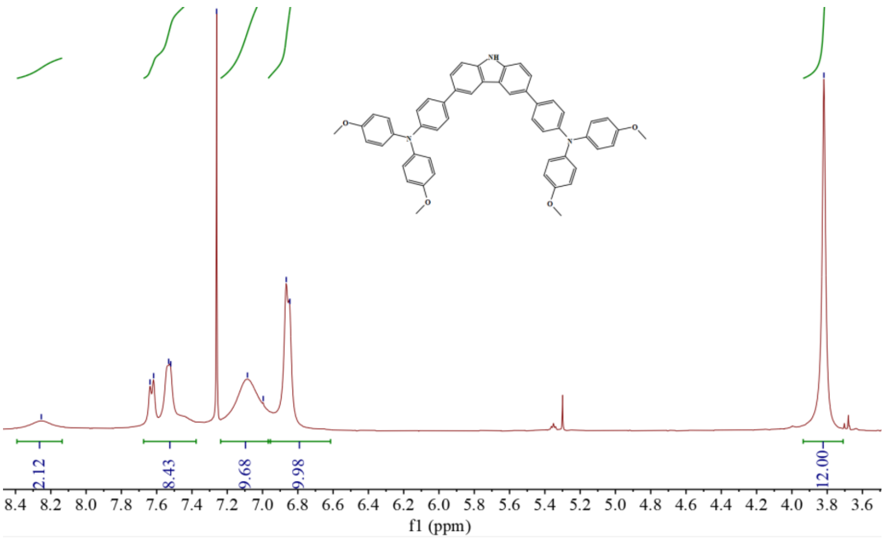

Figure S5. ^{1}H NMR spectra of CZTPA.

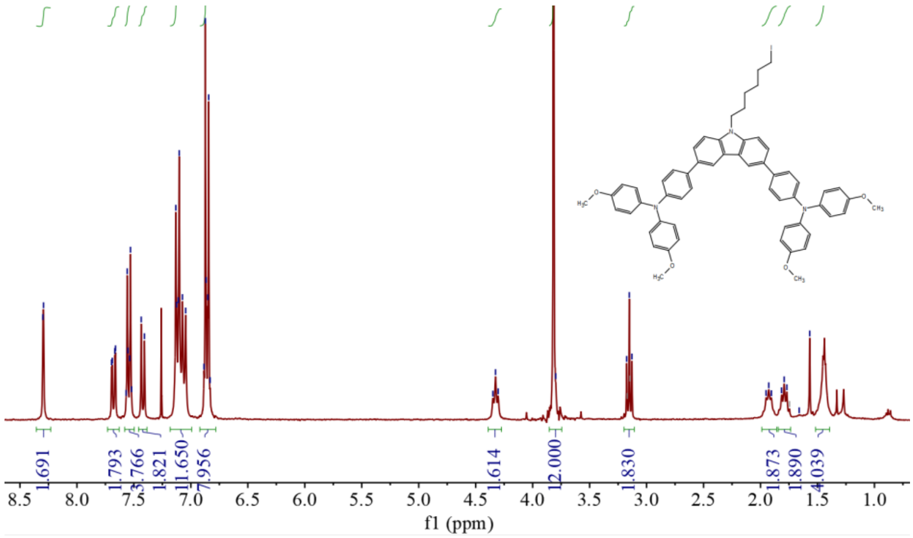

Figure S6. ^{1}H NMR spectra of O1.

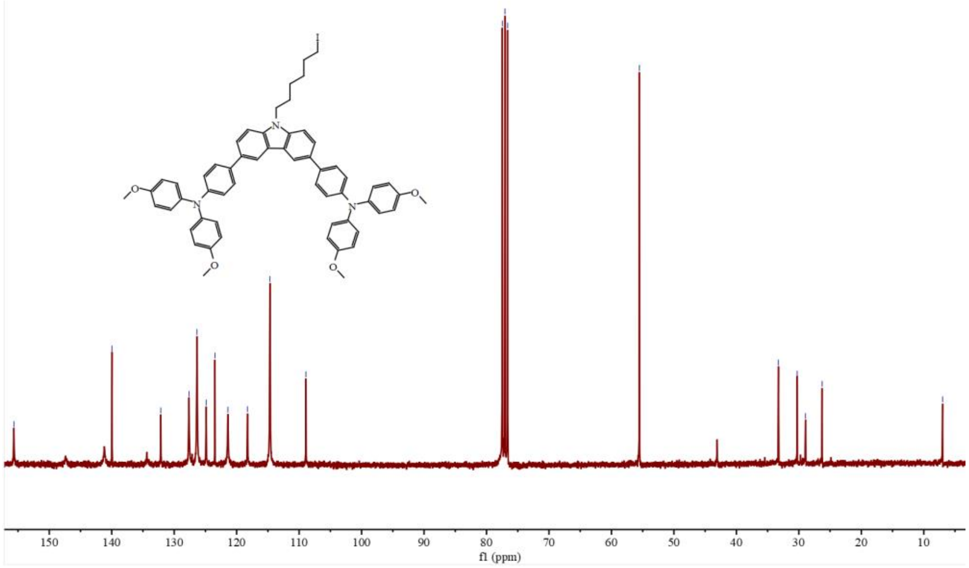

Figure S7. ^{13}C NMR spectra of O1.

2.2 TGA spectra

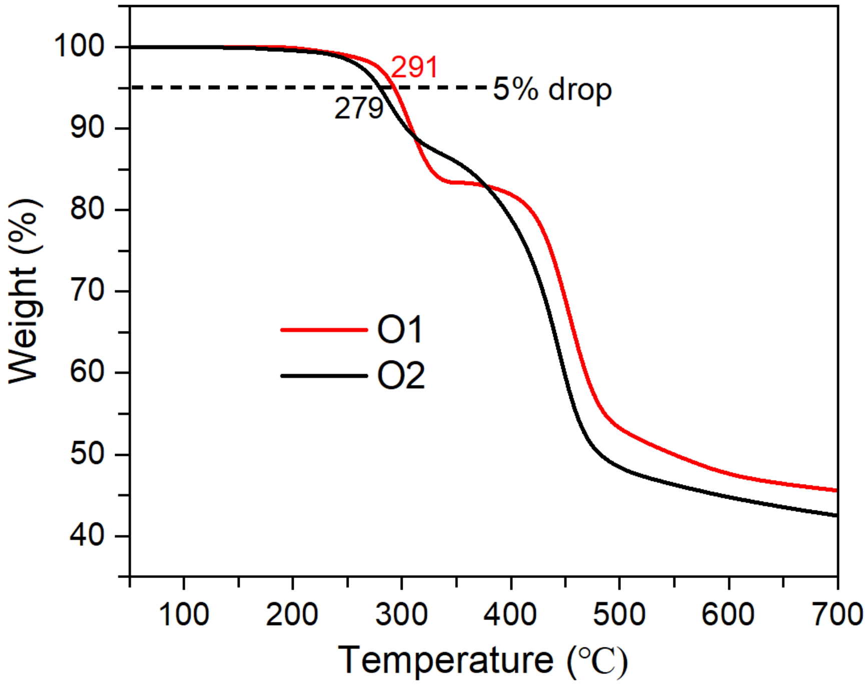

Figure S8. TGA spectra of O1 and O2 molecules.

2.3 Simulated dimer arrangement

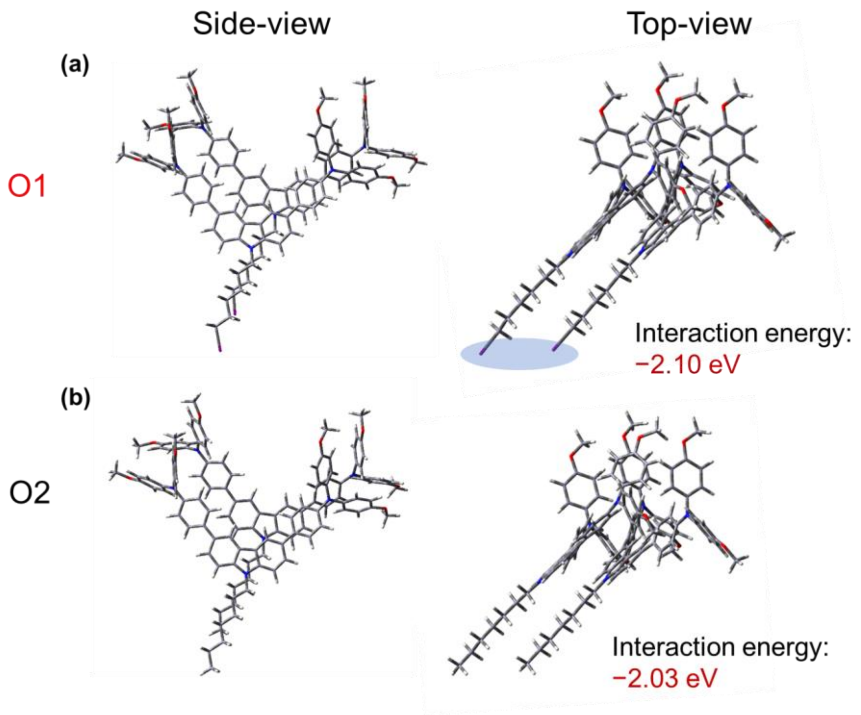

Figure S9. Side view (left) and top view (right) of optimized arrangement of (a) O1 and (b) O2 dimers. The interaction energy within a dimer is highlighted.

2.4 XRD patterns of HTM films

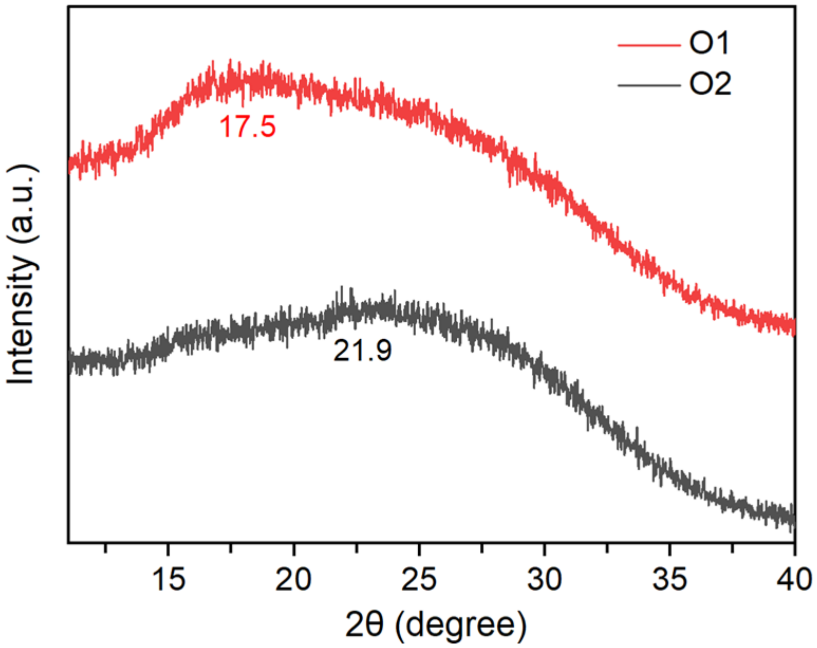

Figure S10. XRD patterns of O1 and O2 films.

2.5 XPS spectra of HTM/perovskite films

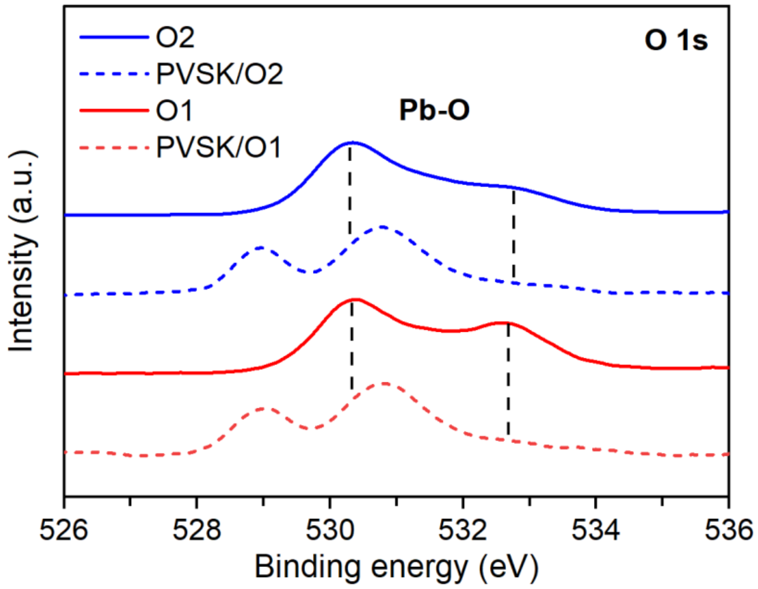

Figure S11. XPS spectra of HTM films with and without coating on the perovskites for O element.

2.6 Steady-state output test

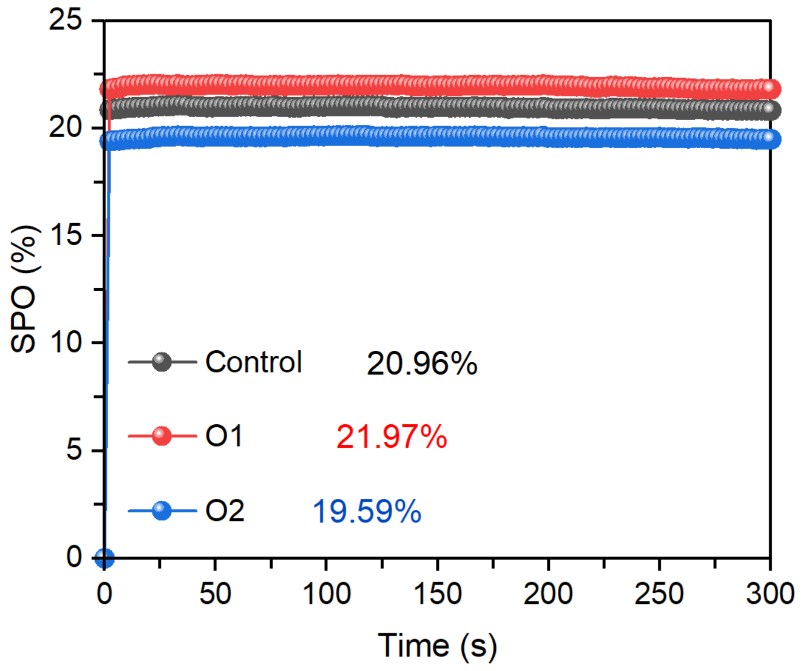

Figure S12. 300 s device steady state output test.

2.7 Dark *J-V* curves of hole-only devices

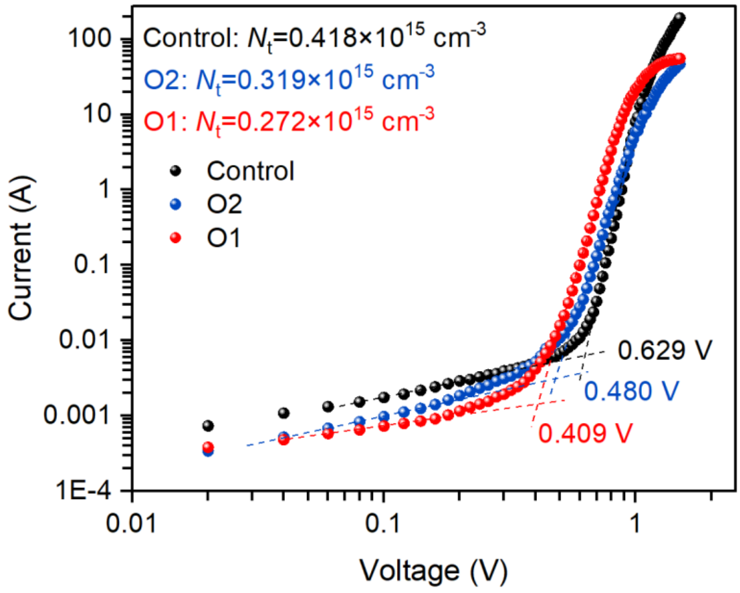

Figure S13. Dark current density (J)–voltage (V) curves of hole-only devices with structure ITO/PEDOT:PSS/PVSK/HTM/Ag with (a) O1, and (b) O2 as the HTMs., respectively. The crossing point of dash lines highlights the kink point V_{TFL}, i.e., trap-filled limit voltage, which allows to calculate the hole trap density (n_{trap}) as follows: $n_{trap} = \frac{2\varepsilon\varepsilon_0 V_{TFL}}{eL^2}$, where e is the elementary charge, L is the thickness of perovskite layer, ε is the relative dielectric constant of perovskite, and ε_0 is the vacuum permittivity.

2.8 WCAs for HTMs coated on glasses

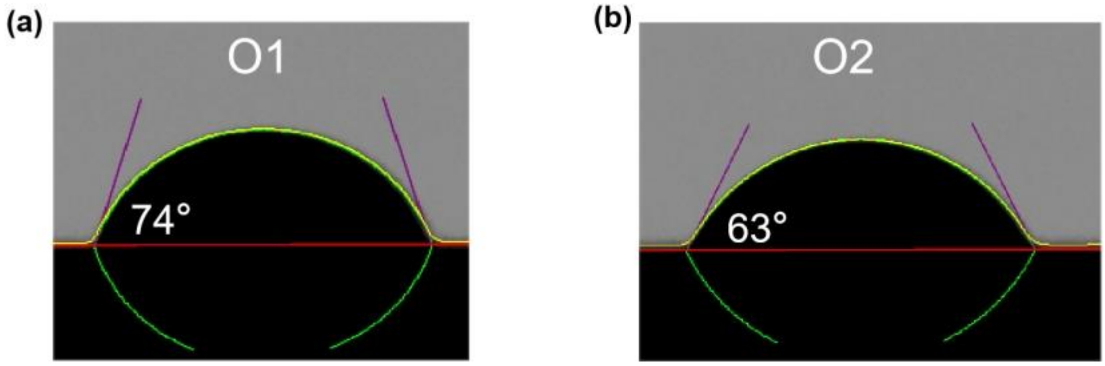

Figure S14. Water contact angles (WCAs) for (a) O1 and (b) O2 HTMs coated on glasses.

References

[1] F. Neese, *WIREs Comput. Mol. Sci.* **2012**, *2*, 73.

[2] C. Bannwarth, S. Ehlert, S. Grimme, *J. Chem. Theory Comput.* **2019**, *15*, 1652.
[3] T. D. Kühne, M. Iannuzzi, M. Del Ben, V. V. Rybkin, P. Seewald, F. Stein, T. Laino, R. Z. Khaliullin, O. Schütt, F. Schiffmann, D. Golze, J. Wilhelm, S. Chulkov, M. H. Bani-Hashemian, V. Weber, U. Borštnik, M. Taillefumier, A. S. Jakobovits, A. Lazzaro, H. Pabst, T. Müller, R. Schade, M. Guidon, S. Andermatt, N. Holmberg, G. K. Schenter, A. Hehn, A. Bussy, F. Belleflamme, G. Tabacchi, A. Glöß, M. Lass, I. Bethune, C. J. Mundy, C. Plessl, M. Watkins, J. VandeVondele, M. Krack, J. Hutter, *J. Chem. Phys.* **2020**, *152*, 194103.
[4] Grimme, S.; Bannwarth, C.; Shushkov, *J. Chem. Theory Comput.* **2017**, *13* (5), 1989–2009.
[5] T. Lu, F. Chen, *J. Comput. Chem.* **2012**, *33*, 580.
[6] T. Lu, *J. Chem. Phys.* **2024**, *161*, 082503.
[7] K. Momma, F. Izumi, *J Appl Crystallogr* **2011**, *44*, 1272.
